# Structure-Enhanced Mechanically Robust Graphite Foam with Ultrahigh MnO_2_ Loading for Supercapacitors

**DOI:** 10.34133/2020/7304767

**Published:** 2020-11-10

**Authors:** Qinghe Cao, Junjie Du, Xiaowan Tang, Xi Xu, Longsheng Huang, Dongming Cai, Xu Long, Xuewen Wang, Jun Ding, Cao Guan, Wei Huang

**Affiliations:** ^1^Frontiers Science Center for Flexible Electronics, Institute of Flexible Electronics, Northwestern Polytechnical University, Xi'an 710072, China; ^2^Department of Materials Science and Engineering, National University of Singapore, 9 Engineering Drive 1, Singapore, Singapore 117576; ^3^College of Chemical Engineering, Hubei University, Wuhan 430062, China; ^4^School of Mechanics, Civil Engineering and Architecture, Northwestern Polytechnical University, Xi'an 710072, China

## Abstract

With the fast bloom of flexible electronics and green vehicles, it is vitally important to rationally design and facilely construct customized functional materials with excellent mechanical properties as well as high electrochemical performance. Herein, by utilizing two modern industrial techniques, digital light processing (DLP) and chemical vapor deposition (CVD), a unique 3D hollow graphite foam (HGF) is demonstrated, which shows a periodic porous structure and robust mechanical properties. Finite element analysis (FEA) results confirm that the properly designed gyroidal porous structure provides a uniform stress area and mitigates potential structural failure caused by stress concentrations. A typical HGF can show a high Young's modulus of 3.18 MPa at a low density of 48.2 mg cm^−3^. The porous HGF is further covered by active MnO_2_ material with a high mass loading of 28.2 mg cm^−2^ (141 mg cm^−3^), and the MnO_2_/HGF electrode still achieves a satisfactory specific capacitance of 260 F g^−1^, corresponding to a high areal capacitance of 7.35 F cm^−2^ and a high volumetric capacitance of 36.75 F cm^−3^. Furthermore, the assembled quasi-solid-state asymmetric supercapacitor also shows remarkable mechanical properties as well as electrochemical performance.

## 1. Introduction

The growing requirement for energy in electronics as well as vehicles has prompted extensive researches on the development of high-performance energy storage devices with higher energy densities and power densities [1–4]. Increasing the mass loading (larger than 10 mg cm^−2^) for commercially available electrodes often leads to the decline of utilization efficiency of the active materials due to sluggish ion/electron transport in bulk reactions [5–11]. Design of a 3D structured electrode containing interconnected porous network can ensure efficient charge transport throughout the entire electrode, which is necessary for the utilization of all active materials and the realization of high rate capability with high capacity/capacitance. With the fast development of 3D printing technology, it has been widely utilized for the construction of functional materials with unique predesigned structures for efficient energy storage devices [12–19]. For example, 3D graphene/graphite-based materials have been widely studied for high-performance energy storage devices due to their low density, high conductivity, and excellent electrochemical stability [20–24]. However, most of the previous studies on 3D printed electrode materials were focused on extrusion-based techniques, where the resolution is low (usually larger than 200 *μ*m) and only certain simple 3D structures (like lattices and interdigitated structure) can be achieved. In addition, the mechanical properties of such 3D carbon materials, which are essential for packaging, transportation, and utilization, are also important for the realization of high-performance energy storage devices, but they were merely discussed in previous reports [11, 15, 18, 24–27]. With the above concern, it will be very promising to develop novel 3D printed electrodes with higher resolution and unique structure design, which will bring about promising mechanical properties and electrochemical performance.

In this work, an ultralight and ultrastiff HGF with a gyroid structure is prepared though integrating DLP technology with the CVD method. Different from extrusion-based 3D printing, DLP technology is based on a fast photopolymerization process where few structural supports are required; thus, various complex 3D architectures with high resolution can be achieved [28–31]. The selected gyroid structure has been reported with high stiffness at low density, but it is hard to construct it with extrusion-based methods [24, 26]. FEA results confirm that the gyroid HGF can provide a uniform stress distribution, thereby mitigating early structural failure caused by stress concentrations. The final HGF can show a high Young's modulus of 3.18 MPa at the low density of 48.2 mg cm^−3^ in compressive performance tests. In addition to the mechanical robustness, the HGF electrode with well-designed hierarchical porosity also can deliver a high mass loading of MnO_2_ with superior electrochemical performance. MnO_2_/HGF with a high mass loading of 28.2 mg cm^−2^ (141 mg cm^−3^) achieves a high areal capacitance of 7.35 F cm^−2^ and a high volumetric capacitance of 36.75 F cm^−3^ with a specific capacitance of 260 F g^−1^. Further assembled HFG-based quasi-solid-state asymmetric supercapacitor also exhibits a remarkable energy density of 0.96 mWh cm^−2^ under a power density of 50 mW cm^−2^ as well as excellent mechanical stability. The 3D HGF with hierarchical porous structure and robust mechanical properties, the customizable facile fabrication process using 3D printing and CVD, together with the promising mechanical and electrochemical properties would pave a good way for the development of high-performance energy storage devices.

## 2. Results

### 2.1. Fabrication of MnO_2_/HGF Electrode

**Figure 1:**
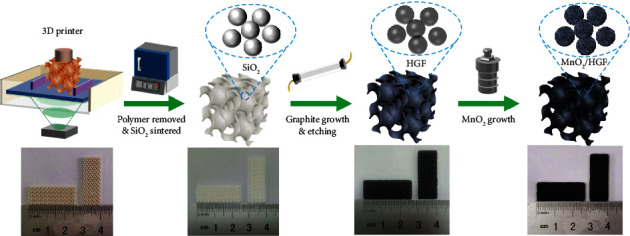
Schematic illustration of the fabrication process of the MnO_2_/HGF electrode.

The schematic fabrication process of the MnO_2_/HGF electrode with the photographs of the samples at each step is shown in Figure 1 (more details are in the Supplementary Materials (available here)). A gyroid SiO_2_ template was firstly designed by computer software and prepared by the high-resolution DLP technology with photopolymerization of UV-curable resin mixed with SiO_2_ microspheres. After 3D printing, a debinding and sintering process was further conducted, in which the photopolymer was completely removed and SiO_2_ was molded. Then, a graphite layer was deposited on the surface of the SiO_2_ template by the CVD method (the experimental setup and reaction route diagram are shown in Figure S1), where the densities and mechanical properties of the graphite can be simply controlled by the deposition time. After etching away the SiO_2_ by aqueous HF solution, MnO_2_ nanosheet arrays were further grown on the surface of HGF, resulting in a hybrid electrode of MnO_2_/HGF.

### 2.2. The Mechanical Properties of HGF

**Figure 2:**
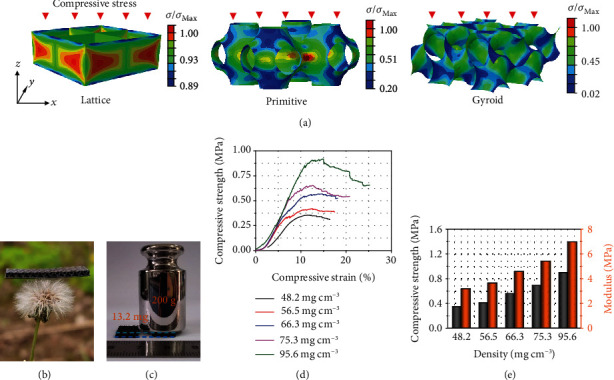
Mechanical properties of HGF. (a) FEA models of lattice, primitive, and gyroid structures and their stress distributions under the same compressive strain along the *z*-direction (the values of *σ*_Max_ for lattice, primitive, and gyroid structure are 600 MPa, 770 MPa and 600 MPa, respectively). (b) Ultralight and (c) Ultrastiff properties illustrated by the gyroidal HGF. (d) Compressive stress-strain curves of HGF with different densities. (e) Compressive strength and Young's modulus of HGF with different densities.

FEA is performed to evaluate the influences of the structure for HGF on their mechanical properties (Figure 2(a)). For comparison, three shell structures including lattice, primitive, and gyroid were modeled and analyzed, and their volume (2 × 2 × 1mm^3^) and thickness (0.1 mm) are fixed consistently. (Red color highlights the stress concentration area. According to the barrel principle, the fracture of these stress concentration points will lead to the destruction of the overall structure.) As indicated from the color distribution, the gyroid structure can provide a uniform stress area with no significant red part. This can be attributed to the continuous periodic structure that can relive stress well, thus effectively avoiding the possible collapse caused by the localized stress concentration. As a comparison, multiple stress concentration points appeared at the connection parts for lattice structure and the neck regions for primitive structure, indicating poor mechanical stability.

In order to further explore the mechanical properties, a gyroidal HGF with an ultralow density of 48.2 mg cm^−3^ was prepared. As demonstrated in Figure 2(b), the HGF with a volume of 2.7∗1∗0.2cm^3^ can steadily stand on a dandelion flower without causing any deformation. More important, the ultralight HGF can withstand 15000 times its own weight without obvious degradation (Figure 2(c)). To further confirm, a compressive test is carried out and the stress-strain responses with different densities under compressive load are recorded in Figure 2(d). A high compressive pressure of 0.35 MPa can be achieved for HGF at a low density of 48.2 mg cm^−3^, and the value increased to 0.9 MPa with a higher density of 95.6 mg cm^−3^. Young's modulus, which can reveal the ability of resisting deformation, is calculated and shown in Figure 2(e). The high values of 3.18 MPa at *ρ* = 48.2mgcm^−3^ and 6.98 MPa at *ρ* = 95.6mgcm^−3^ confirm the high stiffness of HGF, which also stands out from many other carbon-based porous materials, such as graphene-carbon aerogels [32], BC-LRF carbon aerogels [33], graphene crosslinked carbon nanotube sponge/polyimide (Gw-CNT/PI) [34], and carbonaceous aerogels [35]. The FEA and compressive results indicate that the gyroidal HGF can well integrate porous, lightweight properties with promising mechanical stiffness.

### 2.3. Characterization of HGF and MnO_2_/HGF

**Figure 3:**
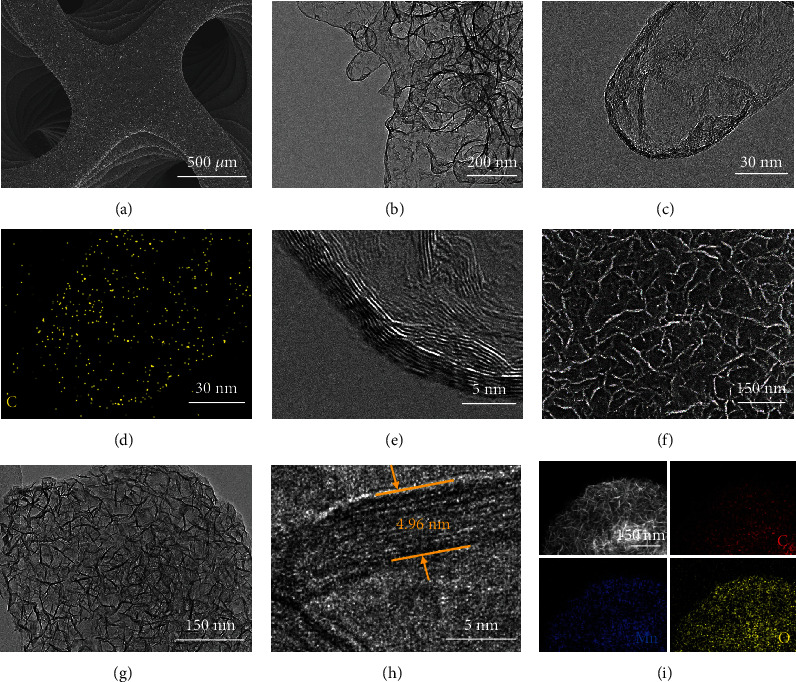
Morphology and structure of the HGF and MnO_2_/HGF: (a) SEM, (b, c) TEM, (d) EDX mapping, and (e) HRTEM images of HGF; (f) SEM, (g) TEM, (h) HRTEM, and (i) EDX mapping images of MnO_2_/HGF.

From the scanning electron microscopy (SEM) image in Figure 3(a), the HGF can well retain the gyroid structure of the SiO_2_ template without any collapse after acid etching, indicating good chemical and structural stability. From enlarged SEM images (Figure S2a and S2b), it can be seen that the HGF has spherical shapes on the surface, which is in accordance with the grain morphology of SiO_2_ before carbon deposition. Transmission electron microscopy (TEM) images in Figures 3(b) and 3(c) illustrate the hollow feature of the HGF after removal of the SiO_2_ template. Similar results can be obtained by energy-dispersive X-ray spectroscopy (EDX) mapping results (Figure 3(d) and Figure S3a). The high-resolution TEM (HRTEM) in Figure 3(e) clearly exhibits regular graphitic lattice fringes, indicating the good crystallinity of HGF. From the Raman spectra in Figure S4, an additional characteristic 2D band peak is detected from HGF (compared to the controlled samples of GO and RGO), revealing the existence of the graphene structure. The X-ray diffraction (XRD) pattern in Figure S5 shows that after acid etching, the diffraction peaks of SiO_2_ completely disappeared and the peaks that appeared at 26.2° and 44.3° belong to the (002) and (101) planes of graphite, respectively.

After a simple hydrothermal reaction, MnO_2_ nanosheets (about 150 nm) are fully covered on the HGF surface (Figure 3(f) and Figure S2c-d). The nanosheets are interconnected to each other to form a network and generate a large number of micropores. Considering the continuous macropores derived by the gyroid structure and the mesopores from graphite microspheres, the MnO_2_/HGF indeed displays hierarchical porosity. Such a hierarchical porous structure provides more accessible surfaces for the electrode-electrolyte contact and reduces the resistance for ion/electron transport. TEM images of MnO_2_/HGF in Figure 3(g) further prove that MnO_2_ nanosheets are uniformly distributed on the HGF surface. From the HRTEM (Figure 3(h)), it can be seen that a typical MnO_2_ nanosheet presents a small sheet thickness of ~4.96 nm, which would fully expose the surface for electrochemical reaction and greatly promote the fast charge transfer within the electrode. The EDX spectrum (Figure S3b) and mapping images (Figure 3(i)) further show the homogeneous element distribution of Mn, O, and C. From the XRD spectrum in Figure S5, the additional two diffraction peaks at 37.1° and 66.7° match well with the (100) and (110) planes of *ε*-MnO_2_ (PDF#30-0802), respectively.

### 2.4. Electrochemical Properties of MnO_2_/HGF

**Figure 4:**
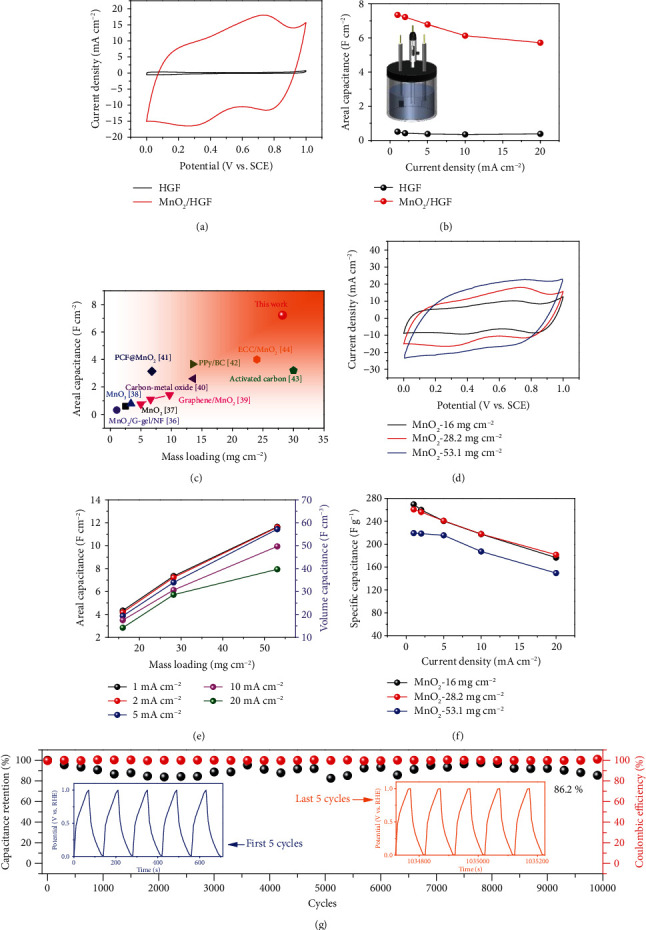
Electrochemical characterizations of MnO_2_/HGF. (a) CV curves and (b) areal capacitances of HGF and MnO_2_/HGF. (c) Comparison of areal capacitance and loading mass of MnO_2_/HGF with those of previously reported MnO_2_-based electrodes. (d) CV curves of MnO_2_/HGF with different mass loadings of MnO_2_. (e) Areal capacitances and volumetric capacitances and (f) specific capacitances obtained from MnO_2_/HGF with different mass loadings of MnO_2_ at different current densities. (g) Cycling performance of MnO_2_/HGF at a current density of 50 mA cm^−2^.

The electrochemical performance of HGF and MnO_2_/HGF electrodes was first studied. As shown in Figure 4(a), after the loading of MnO_2_ nanosheet arrays, the capacitance of HGF is significantly increased with a much larger enclosed area in the cyclic voltammogram (CV) curves. From Figure 4(b) and Figure S6, the bare HGF electrode shows a small capacitance (0.5 F cm^−2^), while the MnO_2_/HGF electrode (with 28.2 mg cm^−2^ of MnO_2_) achieves a high areal capacitance of 7.35 F cm^−2^ at a current density of 1 mA cm^−2^. In addition, the MnO_2_/HGF also demonstrated an excellent high rate capability that was 77.8% of the capacitance that can be maintained when the current density increased from 1 to 20 mA cm^−2^, suggesting highly efficient charge transfer and ion diffusion. To highlight, the MnO_2_/HGF shows promising high mass loading with high areal capacitance, as compared with other MnO_2_-based electrodes [36–44] (Figure 4(c)), showing its great potential for practical usage.

For further optimization, three MnO_2_/HGF electrodes with gradient mass loadings by tuning the hydrothermal reaction time were prepared. As shown in Figure 4(d) and Figure S7, all the three samples show near-rectangular CV behaviors, indicating good reaction kinetics, and the sample with a higher MnO_2_ loading results in a larger enclosed area.  Figure 4(e) further compares the areal, volumetric, and specific capacitances of the three electrodes. The MnO_2_/HGF electrode with 16 mg cm^−2^ of MnO_2_ achieves areal and volumetric capacitances of 4.3 F cm^−2^ and 21.5 F cm^−3^, and the values increase almost linearly to 11.6 F cm^−2^ and 58.2 F cm^−3^, respectively, when the MnO_2_ loading is 53.1 mg cm^−2^. The MnO_2_/HGF electrode with a high MnO_2_ mass loading of 53.1 mg cm^−2^ still maintains 68.2% of the initial capacitance when the current density increased from 1 to 20 mA cm^−2^, demonstrating a superior rate capability. Such observation further proves that the predesigned hierarchical porous carbon structure possesses a large ion/electron accessible area and is beneficial to the rapid charge transfer. In Figure 4(f), it can be seen that the MnO_2_/HGF electrode with 16 mg cm^−2^ of MnO_2_ exhibits a specific capacitance of 269 F g^−1^, and the value decreases slightly to 260 F g^−1^ when the loading mass of MnO_2_ increases to 28.2 mg cm^−2^. Even when MnO_2_ reaches 53.1 mg cm^−2^, the MnO_2_/HGF electrode still achieves a satisfactory specific capacitance of 219.3 F g^−1^. To highlight, the high mass loadings together with the high areal/specific capacitances illustrated by the MnO_2_/HGF compare favorably with the values reported from many other MnO_2_-based electrodes (Table S1) [41, 44, 45]. The obtained electrochemical performance of MnO_2_/HGF can be the result from the gyroid hierarchical porous structure that is beneficial to the transfer of ions/electrons between the electrode and the electrolyte and the well-conducting graphitic carbon with thin-layered MnO_2_ that effectively enhances the reaction kinetics.

The structural merits of HGF are further studied by comparing it with a commonly used carbon cloth (CC) substrate which is highly conductive but has limited porosity (Figure S8). The MnO_2_/CC electrode was prepared using the CC substrate with the same hydrothermal method as MnO_2_/HGF. The mass loading of MnO_2_ can only reach 1.8 mg in MnO_2_/CC, which is only 1/16 of that for MnO_2_/HGF (the thickness of CC is 1/3 of HGF). In addition, the specific capacitance and rate capability of MnO_2_/CC are both poorer than those of MnO_2_/HGF, showing the potential usage of HGF for energy storage devices.

The MnO_2_/HGF electrode with 28.2 mg cm^−2^ of MnO_2_ was further applied for the stability test. As shown in Figure 4(g), at the fixed charge/discharge current density of 50 mA cm^−2^, the MnO_2_/HGF electrode can maintain 86.2% of the initial capacitance after 10000 cycles, demonstrating good cycle stability. SEM images after the cycling test (Figure S9) display the well-maintained structure of MnO_2_/HGF, further confirming the good cycling stability.

### 2.5. HGF-Based Asymmetric Supercapacitor

To prove the practical applications of porous HGF, the negative electrode of polypyrrole/N-doped carbon/HGF (noted as PPy-NC/HGF) is further prepared by electrodepositing PPy on a NC array-coated HGF (Figure S10-12) [46, 47], and an asymmetric supercapacitor based on MnO_2_/HGF and PPy-NC/HGF is assembled (noted as MnO_2_/HGF//PPy-NC/HGF).

The CV curves of the MnO_2_/HGF and PPy-NC/HGF, with a respective potential window of 0 to 1 V and −1 to 0 V, are shown in Figure S13a. The two electrodes can be well-matched with similar capacitance. The CV curves (Figure S13b) of the assembled MnO_2_/HGF//PPy-NC/HGF aqueous supercapacitor show an overall voltage of 0–2 V with good capacitive behavior. As shown in Figure S13c, the GCD curves at various current densities were further evaluated, and an impressive areal capacitance of 2.8 F cm^−2^ is achieved at a current density of 1 mA cm^−2^. When the current density increases to 20 mA cm^−2^, 75% of the value (F cm^−2^) can remain unchanged, indicating the excellent rate capability (Figure S13d). The EIS result (inset in Figure S13d) shows that the aqueous asymmetric supercapacitor possesses small charge transfer resistance and ion diffusion resistance, revealing good electronic conductivity. Two asymmetric supercapacitors connected in series can power sixteen green LEDs and ten blue LEDs (Figure S13e). The MnO_2_/HGF//PPy-NC/HGF also shows good cycling stability (Figure S13e) that 77.7% of the initial capacitance is maintained after 10000 cycles.

**Figure 5:**
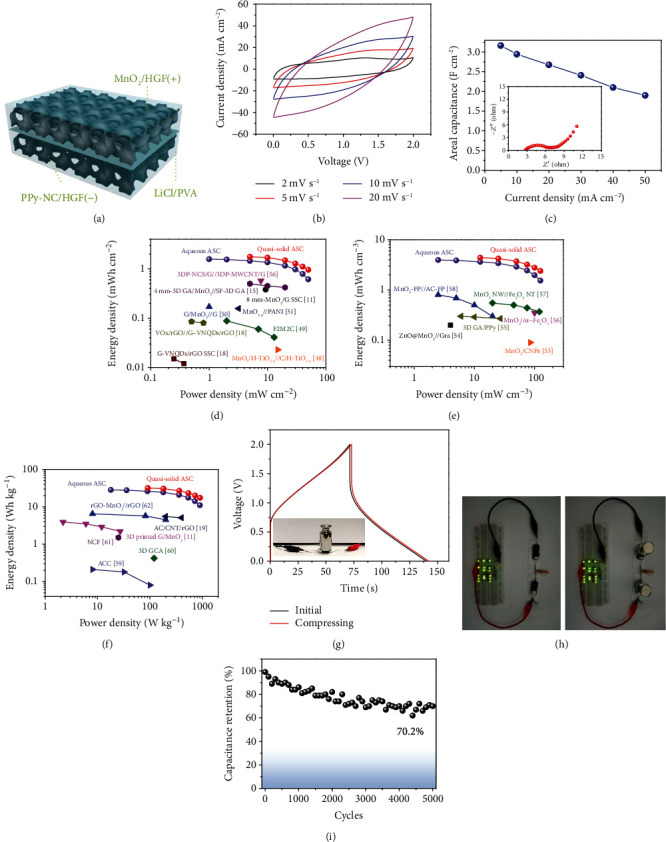
Electrochemical properties of the HFG-based quasi-solid-state supercapacitor. (a) Schematic structure, (b) CV curves, and (c) areal capacitances of the HGF-based asymmetric supercapacitor. Inset in (c) is the EIS results. Ragone plots of aqueous and quasi-solid-state asymmetric supercapacitor based on (d) area, (e) volume, and (f) active material mass of the whole device. (g) Comparison of the CV curves of the asymmetric supercapacitor in its initial state and under compression. (h) Photographs of LEDs powered by two HGF-based supercapacitors in the initial state and under compression. (i) Cycling performance of the HGF-based asymmetric supercapacitor.

A quasi-solid-state asymmetric supercapacitor was also assembled using MnO_2_/HGF and PPy-NC/HGF electrodes with a gel electrolyte, as schematically presented in Figure 5(a). The quasi-solid-state supercapacitor exhibits near-rectangular CV curves with a voltage window of 2 V (Figure 5(b)). Based on the GCD curves (Figure S13f), the quasi-solid-state cell achieves an areal capacitance of 3.165 F cm^−2^ at a current density of 5 mA cm^−2^ and maintains 1.9 F cm^−2^ at a current density of 50 mA cm^−2^, presenting a satisfactory rate capability (Figure 5(c)). Besides, the EIS results (inset of Figure 5(c)) indicate that the quasi-solid-state supercapacitor has small resistance for ion transfer. The areal energy density and high power density of the quasi-solid-state cell are exhibited in Figure 5(d). The MnO_2_/HGF//PPy-NC/HGF cell achieves a high areal energy density of 1.76 mWh cm^−2^ at a power density of 5 mW cm^−2^, and maintains 0.96 mWh cm^−2^ at 50 mW cm^−2^, which are much higher than the other documented works on MnO_2_-based supercapacitors, showing the great promise of the porous HGF (Table S2) [11, 15, 18, 48–52]. The supercapacitor also exhibits a volumetric energy density of 3.94 mWh cm^−3^ at a power density of 2.5 mW cm^−3^ and retains 1.54 mWh cm^−3^ at a high power density of 125 mW cm^−3^ (Figure 5(e)). In addition, it can achieve a specific energy density of 31.9 Wh kg^−1^ at a power density of 90.6 W kg^−1^ and reaches a high power density of 905.8 W kg^−1^ at an energy density of 17.4 Wh kg^−1^ (Figure 5(f)). These values are also better than many previously reported supercapacitors, where the mass loadings are much smaller [11, 19, 53–62].

To further reveal the mechanical robustness of the HGF-based device for practical usage, the GCD curves of the cell in the initial state and under compression were recorded. As shown in Figure 5(g), there is only a slight shift in the GCD curves, indicating its excellent stability under mechanical pressure. One step further, two quasi-solid-state cells connected in series were used to light up 16 green LEDs (Figure 5(h) and Movie 1), and the brightness of the LEDs remains unchanged after the cells are pressed with a heavy counterweight. The quasi-solid-state device also maintains 70.2% of the initial capacitance after 5000 cycles, showing good cycling stability (Figure 5(i)).

## 3. Conclusion

In conclusion, a porous and robust HGF with lightweight has been rationally designed and facilely constructed with the help of DLP and CVD. FEA calculation and compression tests prove that the porous HGF with a gyroidal porous structure can effectively prevent structural failure from stress concentrations thus maintaining mechanical robustness. The graphite foam was further coated with MnO_2_ nanosheets, which can be directly utilized as electrode materials for supercapacitors, without using additional binders and current collectors. Due to the unique hollow and porous structure, not only a high mass loading of active materials can be achieved, the electrode also demonstrated remarkably high areal and volumetric capacitances. A quasi-solid-state asymmetric supercapacitor is further assembled and shows outstanding electrochemical properties as well as excellent mechanical performance. Such a strategy for 3D porous and robust materials with promising mechanical and electrochemical properties would pave a good way for the practical applications of advanced energy storage devices.

## 4. Materials and Methods

The experimental procedure including the preparation of HGF, fabrication of MnO_2_/HGF and PPy-NC/HGF, FEA calculation, characterizations, and electrochemical measurement can be found in the Supplementary Materials.

## Supplementary Materials

Supplementary Materials Figure S1: scheme of experimental setup and reaction route diagram in CVD process. Figure S2: SEM images of HGF and MnO_2_/HGF. Figure S3: EDS of HGF and MnO_2_/HGF. Figure S4: Raman spectra of HGF, rGO, and GO. Figure S5: XRD of HGF/SiO_2_, HGF, and MnO_2_/HGF. Figure S6: electrochemical performance of HGF and MnO_2_/HGF. Figure S7: electrochemical performance of MnO_2_/HGF electrodes with different mass loadings. Figure S8: electrochemical performance of MnO_2_/CC. Figure S9: SEM images of MnO_2_/HGF after 10000-cycle test. Figure S10: SEM images of Co-NC/HGF, NC/HGF, and PPy-NC/HGF and TEM images of PPy-NC/HGF. Figure S11: XRD and Raman spectra of PPy-NC/HGF. Figure S12: electrochemical performance of PPy-NC/HGF. Figure S13: electrochemical performance of aqueous asymmetric supercapacitor. Table S1: summary of the areal capacitance, specific capacitance, and rate capability of electrodes with high mass loading of MnO_2_. Table S2: summary of voltage window, capacitance, and areal energy density (power density) of current study with other reported aqueous and quasi-solid-state devices.
