# Supplementary material for: Structure-Enhanced Mechanically Robust Graphite Foam with Ultrahigh MnO2 Loading for Supercapacitors
Source: Research (Wash D C). 2020 Nov 10;2020:7304767. doi: 10.34133/2020/7304767 (PMC7676245; doi:10.34133/2020/7304767)
Supplement: Supplementary Materials — Figure S1: scheme of experimental setup and reaction route diagram in CVD process. Figure S2: SEM images of HGF and MnO2/HGF. Figure S3: EDS of HGF and MnO2/HGF. Figure S4: Raman spectra of HGF, rGO, and GO. Figure S5: XRD of HGF/SiO2, HGF, and MnO2/HGF. Figure S6: electrochemical performance of HGF and MnO2/HGF. Figure S7: electrochemical performance of MnO2/HGF electrodes with different mass loadings. Figure S8: electrochemical performance of MnO2/CC. Figure S9: SEM images of MnO2/HGF after 10000-cycle test. Figure S10: SEM images of Co-NC/HGF, NC/HGF, and PPy-NC/HGF and TEM images of PPy-NC/HGF. Figure S11: XRD and Raman spectra of PPy-NC/HGF. Figure S12: electrochemical performance of PPy-NC/HGF. Figure S13: electrochemical performance of aqueous asymmetric supercapacitor. Table S1: summary of the areal capacitance, specific capacitance, and rate capability of electrodes with high mass loading of MnO2. Table S2: summary of voltage window, capacitance, and areal energy density (power density) of current study with other reported aqueous and quasi-solid-state devices. [file 7304767.f1.zip › 7304767.f1.docx]

**Supplementary Materials**

Structure Enhanced Mechanically Robust Graphite Foam with Ultrahigh MnO_2_ Loading for Supercapacitors

Qinghe Cao,^1^ Junjie Du,^1^ Xiaowan Tang,^1^ Xi Xu,^2,*^ Longsheng Huang,^3^ Dongming Cai,^3^ Xu Long,^4^ Xuewen Wang,^1^ Jun Ding,^2^ Cao Guan,^1,*^ and Wei Huang^1,*^

^1^Frontiers Science Center for Flexible Electronics, Institute of Flexible Electronics, Northwestern Polytechnical University, Xi’an 710072, P. R. China

E-mail: iamcguan@nwpu.edu.cn, iamwhuang@nwpu.edu.cn.

^2^Department of Materials Science and Engineering, National University of Singapore,

9 Engineering Drive 1, Singapore 117576, Singapore

E-mail: xu_xi@u.nus.edu

^3^College of Chemical Engineering, Hubei University, Wuhan 430062, P. R. China

^4^School of Mechanics, Civil Engineering and Architecture,

Northwestern Polytechnical University, Xi’an 710072, P. R. China

**Experimental section**

*Preparation of SiO_2_* *gyroid foam.*

The preparation process of SiO_2_ gyroid foam is modified based on our previous work, [1] which mainly includes the preparation of SiO_2_ resin, 3D printing and sintering. The SiO_2_ resin was prepared by mixing SiO_2_ powder, Variquat CC 42 NS, photocurable resin and TPO photoinitiator. 3D printing was processed by Asiga MAX 27UV (with a UV source of 385 nm, layer resolution of 27 μm) where the built structure has dimensions of 270 × 10 × 2 mm^3^. In sintering process, the 3D printed SiO_2_ resin template was annealed at 1350 ℃ for 5 h.

*Fabrication of hollow graphite foam (HGF).*

HGF was prepared by a direct CVD route with SiO_2_ gyroid foam serving as the template. The CVD system was first purged with highly purified Ar for 0.5 h. Then the SiO_2_ gyroid foam was heated to 1000 ºC and hold for 1 h. After that, ethanol bubbled by Ar is introduced to the quartz tube for another 0.5-1.5 h at 1000 ºC. After cooling to room temperature, the whole product was immersed in HF aqueous solution overnight to remove the SiO_2_ template. The sample was washed by DI water and freeze-dried with liquid N_2_, after which the final HGF can be obtained.

*Fabrication of MnO_2_/HGF.*

MnO_2_/HGF was prepared by a simple one-step hydrothermal method. In a typical synthetic process, different amount of KMnO_4_, 0.5 mL HCl were dissolved in 35 mL of distilled water and stirred to form a homogeneous solution. Then the solution was transferred into a 50 mL Teflon-lined stainless autoclave. Subsequently, HGF was immersed into the solution and maintained at 85 °C for 20 min. The final mass loading of MnO_2_ was about 16, 28.2 and 53.1 mg cm^-2^ with different amount of 0.158 g, 0.316 g and 0.474 g KMnO_4_ added, respectively.

*Fabrication of MnO_2_/CC.*

The preparation of MnO_2_/CC is similar to the MnO_2_/HGF, where HGF is replaced with carbon cloth.

*Fabrication of PPy-NC/HGF.*

Firstly, 60 mL aqueous solution contains 2-methylimidazole (0.4 M) was quickly added to 60 mL aqueous solution of Co(NO_3_)_2_·6H_2_O (0.05 M), then a HGF was immersed into the solution for 4 h. After that, the obtained Co-MOF/HGF was annealed in N_2_ at 800 °C with a heating rate of 2 °C min^-1^ for 2h. Subsequently, the sample was immersed in 2 M FeCl_3_ solution for overnight to achieve NC/HGF. The PPy-NC/HGF composite was synthesized by electrodeposition of pyrrole on NC/HGF at an applied potential of 0.7 V (vs. Ag/AgCl) in 0.2 M pyrrole and 0.2 M NaNO_3_ aqueous solution for 1 h.

*Fabrication of* *quasi-solid-state asymmetric supercapacitor.*

The quasi-solid-state asymmetric supercapacitor was assembled by using MnO_2_/HGF as positive electrode, PPy-NC/HGF as negative electrode, and PVA/LiCl gel as the electrolyte. The PVA/LiCl gel electrolyte was prepared by dissolving 7.375 g of LiCl and 3 g of PVA power in 30 mL of deionized water at 90 °C. Subsequently, both MnO_2_/HGF and PPy-NC/HGF electrode were soaked in PVA/ LiCl gel electrolyte for 5 min and then solidified at 45 °C for 10 min. Finally, the two electrodes were assembled face to face and the device was left overnight until the electrolyte was solidified.

*FEA calculation.*

The finite element analysis comparing three different 3D structures is implemented in commercial package ABAQUS. All models have the same volume (2*2*1 mm^3^) and thickness (0.1 mm). The elastic properties of the material refer to graphite, whose elastic modulus is 5.85 GPa and Poisson's ratio is 0.07. Linear elastic analysis is performed. The simulation uses the traditional shell unit (S4R) and explicit dynamic analysis. The boundary condition is set to fix the bottom in all directions and the top displacement in all directions except the Z-axis, followed by 0.1 mm z axial displacement in Z-axis to compress the three structures.

*Materials characterization.*

The materials were characterized by XRD pattern (Bruker D8 Advanced) with radiation from a Cu target, field emission scanning electron microscopy (FESEM, FEI Verios G4, 20 kV), transmission electron microscopy (TEM, FEI Talos F200X, 200 KV), Raman spectra (WITec Alpha300R, 532nm), and Compression test (NCS GNT100).

*Compression test.*

The HGF was loaded displacement controlled at a rate of 0.1 mm s^−1^ for 10 kN load cell in the direction of vertical plane until failed. According to the obtained stress-strain curves, the compressive properties of HGF were calculated, where the modulus is the slope of the linear deformation region of the curve. The size of samples is fixed at 1.5*1*0.2 cm^3^.

*Electrochemical measurement.*

Electrochemical measurements including CV curves, galvanostatic charge–discharge (GCD) curves, EIS (the frequency range from 100 kHz to 0.01 Hz) of single electrode, aqueous asymmetric supercapacitor and quasi-solid-state asymmetric supercapacitor were carried out using an electrochemical workstation (CHI 760E, CH Instruments Inc., Shanghai). The cycling performance measurement were carried out using NEWARE battery testing system and LANHE battery testing system. The electrochemical performance of the electrode materials was first performed in a three-electrode system with 1 M Na_2_SO_4_ as electrolyte solution. Electrode materials (10*10*2 mm^3^), Pt foil, and saturated calomel electrode (SCE) electrode were used as the working electrode, counter electrode, and reference electrode, respectively.

Areal capacitance (*C_A_*), volumetric capacitance (*C_V_*) and specific capacitance (*C*) of single electrodes and devices are calculated based on the GCD curves using following equations:

$C_{A}=\frac{I\times t}{\Delta V\times A}$ (1)

$C_{V}=\frac{I\times t}{\Delta V\times V}$ (2)

$C=\frac{I\times t}{\Delta V\times m}$  (3)

where *C_A_*, *C_V_* and *C* are the areal, volumetric and specific capacitance, respectively. *∆V* is the potential window, *I* is the discharge current, *t* is the discharge time, *A* is the geometric area of the working electrode working, *V* is the volume of the electrodes and *m* is the loading mass of the active materials on the electrodes. For the device, *A* is the geometric area of the device, *V* is the total volume of the device and *m* is the total loading mass of the device. Areal energy density (*E_A_*), volumetric energy density (*E_V_*) and specific energy density (*E*) and corresponding power densities (*P_A_*, *P_V_* and *P*) of devices are calculated using the following equations:

$E_{A}=\frac{1000}{2\times3600}\times C_{A}\times{\Delta V}^{2}$ (4)

$P_{A}=\frac{3600\times E_{A}}{t}$ (5)

$E_{V}=\frac{1000}{2\times3600}\times C_{V}\times{\Delta V}^{2}$ (6)

$P_{V}=\frac{3600\times E_{V}}{t}$ (7)

$E=\frac{1000}{2\times3600}\times C\times{\Delta V}^{2}$ (8)

$P=\frac{3600\times E}{t}$ (9)

**Figure S1.** Scheme of experimental setup and reaction route diagram in CVD process.

**Figure S2.** SEM images of (a-b) HGF and (c-d) MnO_2_/HGF.

**Figure S3.** EDS of (a) HGF and (b) MnO_2_/HGF.

**Figure S4.** Raman spectra of HGF, rGO and GO.

**Figure S5.** XRD of HGF/SiO_2_, HGF and MnO_2_/HGF.

**Figure S6.** (a) CV curves of HGF at different scan rates. (b) GCD curves of HGF with different charge/discharge current densities. (c) CV curves of MnO_2_/HGF at different scan rates. (d) GCD curves of MnO_2_/HGF with different charge/discharge current densities.

**Figure S7.** (a) CV curves of MnO_2_/HGF-16 mg cm^-2^ at different scan rates. (b) GCD curves of MnO_2_/HGF-16 mg cm^-2^ with different charge/discharge current densities. (c) CV curves of MnO_2_/HGF-53.1 mg cm^-2^ at different scan rates. (d) GCD curves of MnO_2_/HGF-53.1 mg cm^-2^ with different charge/discharge current densities. (e) Comparation of GCD curves of three MnO_2_/HGF electrodes with different MnO_2_ loading masses at a charge/discharge current density of 1 mA cm^-2^. (f) EIS of the three MnO_2_/HGF electrodes with different MnO_2_ loading masses.

**Figure S8.** (a) CV curves of MnO_2_/CC at different scan rates. (b) GCD curves of MnO_2_/CC with different charge/discharge current densities. (c) Comparation of CV curves of MnO_2_/CC and MnO_2_/HGF at a scan rate of 2 mV s^-2^. (d) Comparation of GCD curves of MnO_2_/CC and MnO_2_/HGF at a charge/discharge current density of 20 mA cm^-2^. (e) Specific capacitance of MnO_2_/CC and MnO_2_/HGF based on their corresponding GCD curves. (f) EIS of MnO_2_/CC and MnO_2_/HGF.

**Figure S9.** SEM images of MnO_2_/HGF after 10000 cycles test.


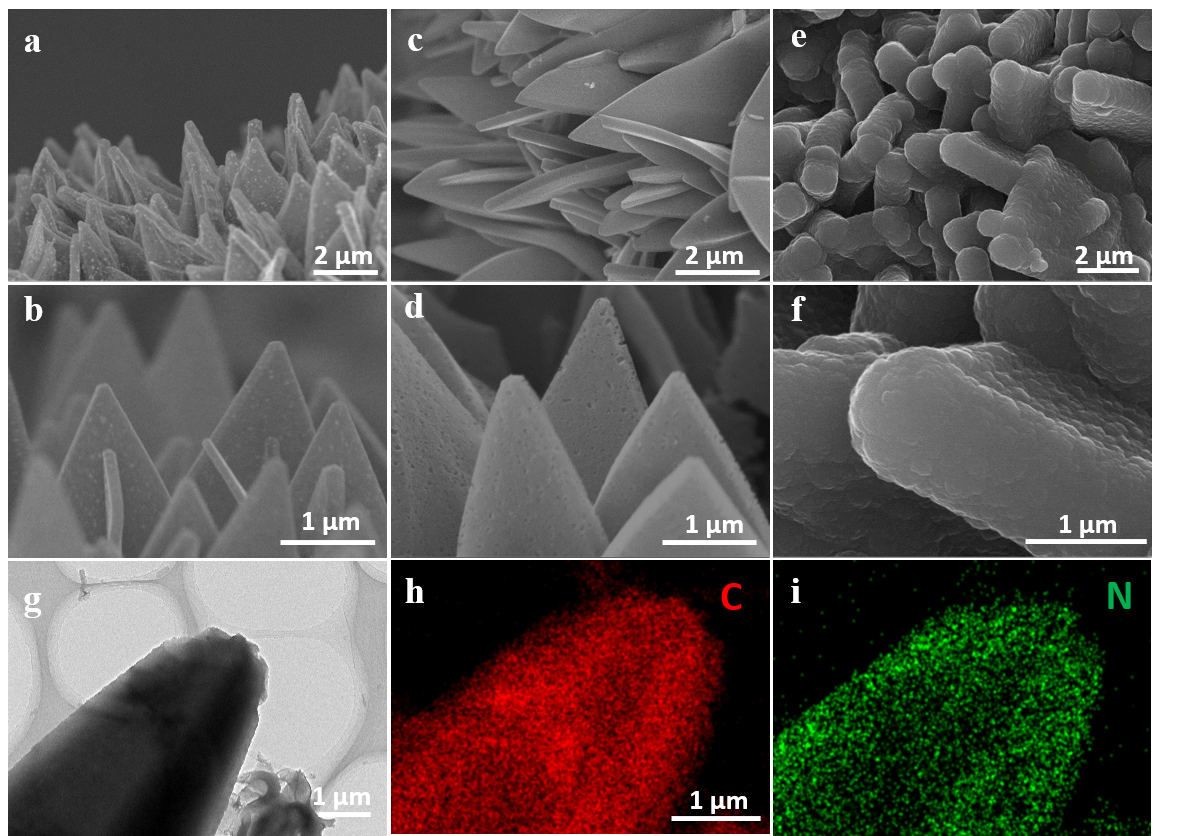


**Figure S10.** SEM images of (a-b) Co-NC/HGF, (c-d) NC/HGF, (e-f) PPy-NC/HGF. (g) TEM image of PPy-NC/HGF. (h-i) TEM mapping results of PPy-NC/HGF. PPy-NC still maintain a good triangular structure regardless of the increasing in thickness of the nanowalls (1 μm) due to the high mass loading of PPy. The EDX mapping proved that C and N are uniform distributed.

**Figure S11.** (a) XRD of NC/HGF and PPy-NC/HGF. (b) Raman spectra of PPy-NC/HGF. It can be concluded that there is no new diffraction peak generated after the deposition of PPy in XRD. Additional peaks occurred at around 921, 989, 1044, and 1406 cm^-1^ in Raman spectrum indicate the successful synthesis of PPy.

**Figure S12.** (a) CV curves of PPy-NC/HGF at different scan rates. (b) GCD curves of PPy-NC/HGF with different charge/discharge current densities. (c) EIS of PPy-NC/HGF. (d) Specific capacitances of PPy-NC/HGF based on their corresponding GCD curves. The synthesized PPy-NC/HGF electrode can exhibit a high capacitive performance, wide potential window of 1 V and good conductivity at high mass loading of 27.2 mg cm^-2^. The PPy-NC/HGF electrode exhibits a good specific capacitance of 190 F g^-1^ at a current density of 1 mA cm^-2^ as well as a superior rate compatibility (72.6% of the capacitance is maintained when the current density increased from 1 to 20 mA cm^-2^).

**Figure S13.** Electrochemical performance of aqueous asymmetric supercapacitor. (a) Comparison of CV curves for MnO_2_/HGF and PPy-NC/HGF electrodes. (b) CV curves of aqueous asymmetric supercapacitor at various scan rates. (c) GCD curves of aqueous asymmetric supercapacitor with different charge/discharge current densities. (d) Areal capacitance of aqueous asymmetric supercapacitor based on their corresponding GCD curves. Inset is the EIS of aqueous asymmetric supercapacitor. (e) Cycling performance of aqueous asymmetric supercapacitor. Inset shows photographs of LEDs powered by aqueous asymmetric supercapacitor. (f) GCD curves of quasi-solid-state asymmetric supercapacitor with different charge/discharge current densities.

**Table S1**. Summary of the areal capacitance, specific capacitance and rate capability of electrodes with high mass loading of MnO_2_.

| Materials | Loading  Mass  (mg cm^-2^) | Electrolyte | Areal Capacitance  (F cm^-2^) | Specific capacitance  (F g^-1^) | Rate  Capability | Ref. |
| --- | --- | --- | --- | --- | --- | --- |
| ECC/MnO2 | 23.5 | 5 M  LiCl | 4.2 F cm^-2^  (5 mV s^-1^) | 118.3  (5 mV s^-1^) | 30%  (5-100 mV s^-1^) | [2] |
| MnO_2_-CNT-textile | 8.3 | 0.5 M Na_2_SO_4_ | 2.8 F cm^-2^  (0.05 mV s^-1^) | -- | 57%  (0.05-0.8 mV s^-1^) | [3] |
| NGMn | 2 | 5 M  LiCl | 0.43 F cm^-2^ (0.25 A g^-1^) | 240  (10 mV s^-1^) | 65.6%  (5-100 mV s^-1^) | [4] |
| GF/CNT/MnO_2_ | 8.4 | 0.5 M Na_2_SO_4_ | 0.73 F cm^-2^  (5 mV s^-1^) | 75  (5 mV s^-1^) | 12%  (5-100 mV s^-1^) | [5] |
| MnO_2_-PEDOT:PSS | 8.5 | 0.5 M Na_2_SO_4_ | 1.67 F cm^-2^ (0.5 mA cm^-1^) | 140 F g^-1^  (2 mV s^-1^) | 8%  (4-100 mV s^-1^) | [6] |
| graphene/MnO_2_ | 9.8 | 0.5 M Na_2_SO_4_ | 1.4 F cm^-2^  (2 mV s^-1^) | 130 F g^-1^  (2 mV s^-1^) | 7%  (2-100 mV s^-1^) | [7] |
| NNA@MnO_2_ | 16.99 | 0.5 M Na_2_SO_4_ | 2.4 F cm^-2^  (1 mV s^-1^) | 120 F g^-1^  (1 mV s^-1^) | -- | [8] |
| MnO_2_/Ni foam | 18 | 1 M  Na_2_SO_4_ | 2.79 F cm^-2^  (2 mA cm^-2^) | 150 F g^-1^  (2 mA cm^-2^) | 30.9%  (2-20 mA cm^-2^) | [9] |
| MnO_2_ | 3.37 | 1 M  LiCl | 0.39 F cm^-2^  (20 mV s^-1^) | 116 F g^-1^  (20 mV s^-1^) | -- | [10] |
| MnO_2_/G-gel/NF | 13.6 | 0.5 M Na_2_SO_4_ | 3.18 F cm^-2^  (1 mV s^-1^) | 234.2 F g^-1^  (1 mV s^-1^) | 31.4%  (1-40 mV s^-1^) | [11] |
| MnO_2_/PCF | 7 | 6 M  KOH | 3.14 F cm^-2^  (10 mV s^-1^) | -- | 62%  (10-1000 mV s^-1^) | [12] |
| MnO_2_@WC | 25 | 1 M  Na_2_SO_4_ | 4.15 F cm^-2^  (1 mA cm^-2^) | 176.8 F g^-1^  (1 mA cm^-2^) | 43.6%  (1-30 mA cm^-2^) | [13] |
| TCC/MnO_2_ | 13 | 0.5 M Na_2_SO_4_ | 1.2 F cm^-2^  (2 mV s^-1^) | 110 F g^-1^  (2 mV s^-1^) | 50%  (2-20 mV s^-1^) | [14] |
| MnO_2_/TCC | 4.5 | 5 M  LiCl | 2.1 F cm^-2^  (4 mA cm^-2^) | -- | 47.6%  (4-15 mA cm^-2^) | [15] |
| 3D printed  Graphene/MnO_2_ | 45.2 | 3 M  LiCl | 11.55 F cm^-2^  (0.5 mA cm^-2^) | 231.9 F g^-1^  (0.5 mA cm^-2^) | 67.7%  (0.5-20 mA cm^-2^) | [16] |
| MnO_2_/HGF | 16 | 1 M  Na_2_SO_4_ | 4.33 F cm^-2^  (1 mA cm^-2^) | 269.5 F g^-1^  (1 mA cm^-2^) | 65.6%  (1-20 mA cm^-2^) | This  work |
| MnO_2_/HGF | 28.2 | 1 M  Na_2_SO_4_ | 7.35 F cm^-2^  (1 mA cm^-2^) | 260.6 F g^-1^  (1 mA cm^-2^) | 69.6%  (1-20 mA cm^-2^) | This  work |
| MnO_2_/HGF | 53.1 | 1 M  Na_2_SO_4_ | 11.64 F cm^-2^  (1 mA cm^-2^) | 219.3 F g^-1^  (1 mA cm^-2^) | 68.5%  (1-20 mA cm^-2^) | This  work |

**Table S2.** Summary of voltage window, capacitance and areal energy density (power density) of current study with other reported aqueous and quasi-solid-state devices.

| Devices | Voltage window | Electrolyte | Capacitance | Energy density  (Power density) | Ref. |
| --- | --- | --- | --- | --- | --- |
| 3DP-NCS/G//3DP-MWCNT/G | 0-1.6 V | 6 M KOH | 0.746 mAh cm^-2^  (10 mA cm^-2^) | 0.574 mWh cm^-2^  (7.71 mW cm^-2^) | [17] |
| 4mm 3D GA/MnO_2_//SF-3D GA | 0-2 V | 3 M LiCl | 0.82 F cm^-2^  (5 mA cm^-2^) | 0.5 mWh cm^-2^  (5.1 mW cm^−2^) | [18] |
| VOx/rGO//G–VNQDs/rGO | 0-1.6 V | LiCl/PVA | 0.21 F cm^-2^  (6 mA cm^-2^) | 0.076 mWh cm^-2^  (0.5 mW cm^−2^) | [19] |
| 8 mm-MnO_2_/G SSC | 0-0.8 V | 3 M LiCl | 4.5 F cm^-2^  (1 mA cm^-2^) | 0.39 mWh cm^-2^  (9.3 mW cm^−2^) | [16] |
| F2M2C | 0-1 V | 6 M KOH | 2.6 F cm^-2^  (2 mV s^-1^) | 0.057 mWh cm^-2^  (16.3 mW cm^−2^) | [20] |
| AC/CNT/rGO//AC/CNT/rGO | 0-1 V | 6 M KOH | 4.56 F cm^-2^  (5 mV s^-1^) | 0.63 mWh cm^-2^  (10 mW cm^−2^) | [21] |
| Au/rGO/MnO_2_//Au/rGO/MnO_2_ | 0-0.8 V | LiCl/PVA | 1.53 F cm^-2^  (1.5 mA cm^-2^) | 0.136 mWh cm^-2^  (9.58 mW cm^−2^) | [22] |
| AC/CNT/MXene-N/GO | 0-0.6 V | 3 M H_2_SO_4_ | 8.2 F cm^-2^  (10 mV s^-1^) | 0.42 mWh cm^-2^  (8.2 F cm^-2^) | [23] |
| F-GRF//F-GRF | 0-1 V | 6 M KOH | 3.8 F cm^-2^  (2 mV s^-1^) | 0.52 mWh cm^-2^  (3.7 mW cm^−2^) | [24] |
| MnO_2_/CNT//MoS_2_/CNT | 0-1.8 V | LiCl/PVA | 0.42 F cm^-2^  (25 mV s^-1^) | 0.198 mWh cm^-2^  (18.6 mW cm^−2^) | [25] |
| rGO-Co_3_O_4_//rGO | 0-1.4 V | KOH/PVA | 0.14 F cm^-2^  (2 mA cm^-2^) | 0.036 mWh cm^-2^  (1.38 mW cm^−2^) | [26] |
| NiCoP//AC | 0-1.6 V | 2 M KOH | 3.26 F cm^-2^  (2 mA cm^-2^) | 1.16 mWh cm^-2^  (1.6 mW cm^−2^) | [27] |
| MnO_2_/HGF/PPy-NC/HGF | 0-2 V | LiCl/PVA | 3.165 F cm^-2^  (5 mA cm^-2^) | 1.76 mWh cm^-2^  (5 mW cm^-2^),  0.96 mWh cm^-2^  (50 mW cm^-2^) | This  work |

**Reference**

[1] X. Xu, C. Guan, L. Xu et al., "Three Dimensionally Free-Formable Graphene Foam with Designed Structures for Energy and Environmental Applications," *ACS Nano*, vol. 14*,* no. 1, pp. 937-947, 2020.

[2] Y. Song, T. Liu, B. Yao et al., "Ostwald Ripening Improves Rate Capability of High Mass Loading Manganese Oxide for Supercapacitors," *ACS Energy Letters*, vol. 2*,* no. 8, pp. 1752-1759, 2017.

[3] L. Hu, W. Chen, X. Xie et al., "Symmetrical MnO_2_-Carbon Nanotube-Textile Nanostructures for Wearable Pseudocapacitors with High Mass Loading," *ACS Nano*, vol. 5*,* no. 11, pp. 8904-8913, 2011.

[4] Y. Liu, X. Miao, J. Fang et al., "Layered-MnO_2_ Nanosheet Grown on Nitrogen-Doped Graphene Template as a Composite Cathode for Flexible Solid-State Asymmetric Supercapacitor," *ACS Applied Materials & Interfaces*, vol. 8*,* no. 8, pp. 5251-5260, 2016.

[5] J. Liu, L. Zhang, H. B. Wu et al., "High-performance flexible asymmetric supercapacitors based on a new graphene foam/carbon nanotube hybrid film," *Energy & Environmental Science*, vol. 7*,* no. 11, pp. 3709-3719, 2014.

[6] Z. Su, C. Yang, C. Xu et al., "Co-electro-deposition of the MnO_2_–PEDOT:PSS nanostructured composite for high areal mass, flexible asymmetric supercapacitor devices," *Journal of Materials Chemistry A*, vol. 1*,* no. 40, pp. 12432-12440, 2013.

[7] Y. He, W. Chen, X. Li et al., "Freestanding Three-Dimensional Graphene/MnO_2_ Composite Networks As Ultralight and Flexible Supercapacitor Electrodes," *ACS Nano*, vol. 7*,* no. 1, pp. 174-182, 2013.

[8] C. Xu, Z. Li, C. Yang et al., "An Ultralong, Highly Oriented Nickel-Nanowire-Array Electrode Scaffold for High-Performance Compressible Pseudocapacitors," *Advaned Materials*, vol. 28*,* no. 21, pp. 4105-4110, 2016.

[9] J. Yang, L. Lian, H. Ruan, F. Xie and M. Wei, "Nanostructured porous MnO_2_ on Ni foam substrate with a high mass loading via a CV electrodeposition route for supercapacitor application," *Electrochimica Acta*, vol. 136*,* pp. 189-194, 2014.

[10] C. Zhu, L. Yang, J. K. Seo et al., "Self-branched α-MnO_2_/δ-MnO_2_ heterojunction nanowires with enhanced pseudocapacitance," *Materials Horizons*, vol. 4*,* no. 3, pp. 415-422, 2017.

[11] T. Zhai, F. Wang, M. Yu et al., "3D MnO_2_-graphene composites with large areal capacitance for high-performance asymmetric supercapacitors," *Nanoscale*, vol. 5*,* no. 15, pp. 6790-6796, 2013.

[12] T. Liu, Z. Zhou, Y. Guo, D. Guo and G. Liu, "Block copolymer derived uniform mesopores enable ultrafast electron and ion transport at high mass loadings," *Nature Communications*, vol. 10*,* no. 1, pp. 675-685, 2019.

[13] C. Chen, Y. Zhang, Y. Li, Dai et al., "All-wood, low tortuosity, aqueous, biodegradable supercapacitors with ultra-high capacitance," *Energy & Environmental Science*, vol. 10*,* no. 2, pp. 538-545, 2017.

[14] H. Wang, C. Xu, Y. Chen and Y. Wang, "MnO_2_ nanograsses on porous carbon cloth for flexible solid-state asymmetric supercapacitors with high energy density," *Energy Storage Materials*, vol. 8*,* pp. 127-133, 2017.

[15] M. Nakayama, S. Osae, K. Kaneshige, K. Komine and H. Abe, "Direct Growth of Birnessite-Type MnO_2_ on Treated Carbon Cloth for a Flexible Asymmetric Supercapacitor with Excellent Cycling Stability," *Journal ofThe Electrochemical Society*, vol. 163*,* no. 10, pp. A2340-A2348, 2016.

[16] B. Yao, S. Chandrasekaran, J. Zhang et al., "Efficient 3D Printed Pseudocapacitive Electrodes with Ultrahigh MnO_2_ Loading," *Joule*, vol. 3*,* no. 2, pp. 459-470, 2019.

[17] X. Tang, C. Zhu, D. Cheng et al., "Architectured Leaf-Inspired Ni_0.33_Co_0.66_S_2_/Graphene Aerogels via 3D Printing for High-Performance Energy Storage," *Advanced Functional Materials*, vol. 28*,* no. 51, article 1805057, 2018.

[18] B. Yao, S. Chandrasekaran, H. Zhang et al., "3D-Printed Structure Boosts the Kinetics and Intrinsic Capacitance of Pseudocapacitive Graphene Aerogels," *Advanced Materials*, vol. 32*,* no. 8, article e1906652, 2020.

[19] K. Shen, J. Ding and S. Yang, "3D Printing Quasi-Solid-State Asymmetric Micro-Supercapacitors with Ultrahigh Areal Energy Density," *Advanced Energy Materials*, vol. 8*,* no. 20, article 1800408, 2018.

[20] L. Dong, C. Xu, Y. Li et al., "Simultaneous Production of High-Performance Flexible Textile Electrodes and Fiber Electrodes for Wearable Energy Storage," *Advanced Materials*, vol. 28*,* no. 8, pp. 1675-1681, 2016.

[21] T. Gao, Z. Zhou, J. Yu et al., "3D Printing of Tunable Energy Storage Devices with Both High Areal and Volumetric Energy Densities," *Advanced Energy Materials*, vol. 9*,* no. 8, article 1802578, 2019.

[22] A. Morag, J. Y. Becker and R. Jelinek, "Freestanding Gold/Graphene-Oxide/Manganese Oxide Microsupercapacitor Displaying High Areal Energy Density," *ChemSusChem*, vol. 10*,* no. 13, pp. 2736-2741, 2017.

[23] L. Yu, Z. Fan, Y. Shao et al., "Versatile N‐Doped MXene Ink for Printed Electrochemical Energy Storage Application," *Advanced Energy Materials*, vol. 9*,* no. 34, article 1901839, 2019.

[24] L. Sheng, J. Chang, L. Jiang et al., "Multilayer-Folded Graphene Ribbon Film with Ultrahigh Areal Capacitance and High Rate Performance for Compressible Supercapacitors," *Advanced Functional Materials*, vol. 28*,* no. 21, article 1800597, 2018.

[25] H. Peng, G. Qian, N. Li et al., "Flexible Asymmetric Supercapacitors with Ultrahigh Energy Density through Synergistic Design of Electrodes," *Advanced Science*, vol. 5*,* no. 11, article 1800784, 2018.

[26] D. Ghosh, J. Lim, R. Narayan and S. O. Kim, "High Energy Density All Solid State Asymmetric Pseudocapacitors Based on Free Standing Reduced Graphene Oxide-Co_3_O_4_ Composite Aerogel Electrodes," *ACS Applied Materials & Interfaces*, vol. 8*,* no. 34, pp. 22253-22260, 2016.

[27] M. Kong, Z. Wang, W. Wang et al., "NiCoP Nanoarray: A Superior Pseudocapacitor Electrode with High Areal Capacitance," *Chemistry – A European Journal*, vol. 23*,* no. 18, pp. 4435-4441, 2017.
